# Supplementary material for: A service evaluation of clinicians’ Signposting of asylum seekers and refugees (ASRs) attending an emergency department in South-West England
Source: PLOS Glob Public Health. 2026 Feb 12;6(2):e0005748. doi: 10.1371/journal.pgph.0005748 (PMC12900349; doi:10.1371/journal.pgph.0005748)
Supplement: S1 Appendix — (PDF) [file pgph.0005748.s001.pdf]

Question	Raw Data	Code Categories	Codes
Please briefly share why you feel we should or shouldn't ask about immigration status	May not change what i offer or do for them<br><br>I believe healthcare to be a human right<br><br>I always feel everyone is entitled to free healthcare, and I wouldn't like to assume if someone is an refuge, asylum seeker etc<br>If we know their immigration status we could sign post helpful resources / primary secondary care if we facilitate that. In terms of treatment I'm not sure it would make a difference in the ED department.<br>Everyone is entitled to free a&e care regardless. But may open lines of communication if they are stressed about it<br><br>They may need additional help or signposting. May not have access to levels of support afforded to others. May not have a GP<br>Their immigration statues doesn't change the medical needs or treatment, what its important its thier to know their living condition<br><br>Potential to create barriers towards those from other countries<br>I would be concerned about offending people<br>Everyone should be entitled to emergency carr in the UK regardless of the immigration status.<br>Not sure, but if there is legislation regarding access to healthcare dependant on immigration status, then it may be necessary to ask so that we are processing these individuals correctly. Asking these questions may also prove helpful with regards to safeguarding/trafficking etc...<br><br>It should be something we are comfortable to discuss<br><br>True emergency care should be for all<br><br>Only to help understand their issues and provide better guidance and signposting<br><br>Not entirely sure but perhaps if we asked them we could more effectively sign post or help them use services available to them.<br>I don't ask about status in case the NHS would den care or charge patients that can't afford care<br><br>In the emergency department, we should resist any efforts to turn us into border force agents. We are here to care for every patient regardless of their circumstances. I will not engage with the sections of the hospital seeking to penalise people for accessing healthcare<br>We provide emergency care for anyone who requires it no matter their status.<br><br>Asking might improve referrals/access to relevant services and support. We probably don't need to ask everyone<br><br>Emergency Care should be free to everybody regardless of status in my eyes.<br>Could be asked when booking in but shouldn't influence care given therefore not relevant to med history<br>Not relevant for emergency care. If someone were to be admitted and become liable for a large finacial bill they should know so I have occasionally recommended to people they check what is covered. Some people e.g. on holiday without insurance or a reciprocal arrangement prefer a plaster for a broken bone and home than a UK operation and a bill.<br>Not our business to check their status. We can obtain relevant medical history that may require foreign travel.<br>Often likely irrelevant to presentation<br><br>It can make a difference with ongoing referrals. I will be more likely to sort more investigations/treatment in the ED rather than defer<br><br>Local populations I.e common migrants in London, less common in Bristol<br>Person dependant<br><br>Only if clinically relevant<br>Depends whether you think it's relevant ie there other safeguarding issues for example that pertain to their immigration status eg exploitation<br>Asking every patient with the follow-up patient comments (why are you asking etc) would probably only take 1-2 minutes a patient. 300 patients a day. ~5 hours. Half a day of a receptionist.<br>Emergency care should come first	Rights and Equity	Human right (I)
			Universal entitlement (VI)
			Inappropriate for clinical staff (I)
		Change inertia	Lack of change(II)
			Relevancy (V)
		Fear of patient offence	Fear of offence (I)
			racial barriers (I)
		Resource constraints	Time-consuming (I)
			Labour intensive (I)
		Social circumstances	Living conditions (I)
			Safeguarding (I)
		Effect on Ongoing care	Referral (III)
			Signposting (V)
			Financial (II)
Please share any additional comments about GP registration and this community and any suggestions for improving registration	As far as I am aware it is very difficult to register with a GP regardless of status. I'm not sure how this can be improved/monitored<br><br>Improving our education<br><br>Migrants should be assisted with GP enrolement once set up in accommodation<br><br>Those staying at Holiday Inn apparently have access to GP services, but don't seem to be aware of it?? Would be really useful if we could refer them to routine GP care as language line etc is unreliable and time consuming, especially at night and for non emergency issues.<br>Easy to access information for staff about where and how people can register with a GP would be good as well as teaching sessions (we had a really good previous teaching session from a GP in Montpelier)<br><br>I believe it is difficult to register with a GP, even for UK citizens so i suspect it would be difficult for non UK citizens.<br><br>Written advice in multiple languages that can be given from ED<br>I recommend 111 for most of these sort of questions. Hopefully they know more detail than me.<br>They must have access to primary care - prisoners do, why can't we treat this group of people humanely?<br>Nil<br><br>I think lack of awareness of what they are entitled to means many are not registered with a GP or don't know how/when to access GP care. I suspect language is often a barrier when trying to navigate GP telephone lines.<br><br>Improved support from social care<br><br>Registration should be provided within Hotels e.g. Winford Manor, various Holiday Inns	Resource constraints	Social care and support (II)
			Accommodation (II)
			Primary care (III)
		Cultural and language barriers	Difficulty navigating NHS (I)
			Lack of awareness (I)
			Language barrier (I)
		Suggested interventions (also included within relevant questions)	Clinician teaching (I)
			Patient information leaflets (I)
			Referral pathway (I)
Please share any additional comments about your experiences of language and/or cultural barriers with patients	I find language line brilliant.<br><br>I often use google translate as you have to be quick in triage/streaming so time constraints mean I don't use language live as much.<br><br>I am cautious using relatives to translate as I can't be sure they are saying the right things/giving the pt the right info.<br><br>When working at the front desk I find that some patients with different cultural backgrounds come in with quite minor issues that do not need to be in ED and are quite angry/confused when we try redirect them.<br>I find I have access to interpreters but community services don't always. It's a barrier to accessing support for patients.<br><br>Very difficult to get some languages eg Farsi out of hours and not an ideal group to use 'friends' as interpreters but very time pressured overnight and often minor health issues. Very difficult to explore subjects such as exploitation/substance abuse/FGM in ED OOH environment- would be much better at GP, but no signposting.<br><br>Challenging and time consuming! Frustrating as often not able to meet their expectations but I feel a lot of sympathy. Helpful to know what services are available e.g. Haven<br><br>Cultural differences can affect patient expectations but not the care we can offer in ED<br><br>They are frequently very scared and do not wish to share information as they feel that it could be used against them. It is difficult to gain their trust.<br><br>I would not change the care I give to someone who was culturally different from me, but I would bear in mind their/ask about their beliefs to ensure care that suits them/more likely to comply with.<br>"If they speak fairly good English but I want to be clear they have understood what I've said I'm likely to use DeepL (or if language is not covered then I will use Google translate although it's not as good as DeepL). If. Their English is very poor then I find relatives/friends who can translate can be good to get an initial understanding of the presenting complaint. I will then usually use language line for the consultation (history/examination/explanation). I often am more thorough/ try to address most of their problems as I feel they may not seek follow up with their GP (especially if not already registered) in a timely manner.I suspect they are more likely to have delayed presentations and less likely to use services due to not feeling entitled to or not being aware of their existence"	Cultural and language barriers	Perceived over-reaction to advice (I)
			Frustration (I)
			Patient expectations (I)
			Fear of sharing information (I)
			Inappropriate referral to ED due to language barrier (I)
		Negative experiences with translation services	Language line (I)
			Family/friends (II)
		Positive experiences with translations services	Language line (I)
			DeepL (I)
		Resource constraints	Inappropriate environment for complex issues (I)
			Time-restraint (II)
Have you got any suggestions on how education around this topic could be delivered?	An education day so people have to be aware<br><br>"Handover - making nurses aware Formal training daysLeaflets for patients "<br>In house sessions on shift<br><br>Formal training as posters dont get lookled and not everyone can use apps quickly.<br><br>Posters in the department, staff room and FB, huddle meetings discussions.<br><br>Brief education sessions in morning /info on My Emergency app<br>Posters around the dept<br><br>"Early morning teaching. MY ED app resources. f2f sessions. Re Q33 - I think info packs for patients (potentially challenging due to language - would need to be printed in several languages) AND online referral forms to local service would benefit, but am unable to check more than one box!"<br><br>A mixture of deliveries are lways good. Online, F2F<br><br>Clinical Governance<br>As part of ED teaching or early morning nursing teaching. The results of this survey would be very welcome at ED clinical governance (email fiona.murphy@nbt.nhs.uk)<br>Personal experience/stories?<br><br>Having clear information in one document on My Emergency Department app<br><br>"Make it a topic of the week/monthPut it on MyED"<br>Would love a teaching session on this topic - ashamed to admit I hardly know anything about it at all, and given global human movement/displacement is so common these days, it's definitely something all HCPs should have knowledge of!<br>"Via formal and informal teaching.Posters around the departmentOnline and ED SHORTS modules"<br>"Departmental TeachingInduction video to access in own timeMy ED"<br>MyEmergency Department guideline<br>Posters, bite size teaching<br><br>online teaching at your own time<br><br>My ED app, departmental teaching and or emodule<br><br>Brief introduction to available resources, delivered during education/departmental meetings for nurses and doctors, every 4 months or so	Formal teaching	Medical/nursing formal teaching (X)
			Topic of the week (II)
			short/regular teaching sessions (VI)
		Education resources	Posters (IV)
			Social media (I)
			Self directed teaching (II)
			E-module(I)
		Guidance	My ED App (VI)
			Single Document (I)
			Clinical governance (I)
Are there any other ways you feel we could improve our signposting for asylum seeker or refugee patients to other local services?	Information packs and making staff more aware of services<br>Leaflets in different languages<br><br>provide leaflets or small cards for reference in different languages such as Arabic.<br><br>Leaflets to give in various languages/QR codes for them to scan<br>If I had more info about local services I could signpost better<br><br>by making links to printable resources for clinicians so we can go online and print of the up to date information for the service we need. Paper copies go out of date.<br><br>At triage/in the waiting room<br>Some written signposting to local services for patients<br><br>Posters in the waiting room?<br>Leaflets on discharge (need to be multiple language options)<br><br>Posters, improving awareness<br><br>If we ask for it at reception when booking in there could be some resources in waiting room that streaming/receptionists can signpost to<br>"QR codes/ pt information packs"<br><br>Social worker based in ED	Clinician knowledge	Staff awareness of local services (II)
			Link worker (I)
		Patient Resources	Information packs/leaflets (VII)
			Multilingual resources (IV)
			Posters (I)
Are there any other ways you feel we could improve the quality of care we provide to asylum seeker or refugee patients?	It's not usually something that you hear much about, I think educating nurses and making everyone more aware of asking the question because patients don't often tell you<br>Education to staff<br><br>Being more open in assessing whether pts belong to these categories, and thereby being able to offer guidance.<br><br>I've no idea how you support them so can't say<br><br>Teaching all staff on refugees in general and just how miserable and challenging their journey has been to get them to where they are now. Given the barrage of right wing rhetoric on the 'immigration crisis', people need re-education so they can show genuine compassion to these people who are seeking healthcare<br><br>By being more aware of the issues<br>Dedicated pathway to refer for onwards care<br><br>As above<br><br>"Ensure we're documenting numbers and follow up with them if they felt care good/bad. Provide more groups/social interaction Possibly a specific role in ED/trust to be an advocate for this group of patients (who will suffer health inequality and likely to have more psychological trauma/complex needs)"<br>It would be useful to have departmental leaflets available in other languages for us to download and print as needed.<br><br>Not above any other patient group	Formal Teaching	Teaching (II)
		Education Resources	Awareness of health needs/barriers (II)
			Communication skills (I)
			Link worker (I)
		Guidance	Referral guidance (II)
		Evaluation	Recording numbers of ASR patients (I)
			Patient feedback (I)

Patient factors	Clinician factors	Environment/system factors	Interventions
• Fear of patient offence<br>• Social circumstances<br>• Cultural and language barriers	• Negative experiences with translation services<br>• Clinician knowledge<br>• Positive experiences with translations services	• Change inertia<br>• Resource constraints<br>• Effect on Ongoing care	• Formal teaching<br>• Education resources Guidance<br>• Evaluation<br>• Clinician knowledge<br>• Patient resources
