# Supplementary material for: A service evaluation of clinicians’ Signposting of asylum seekers and refugees (ASRs) attending an emergency department in South-West England
Source: PLOS Glob Public Health. 2026 Feb 12;6(2):e0005748. doi: 10.1371/journal.pgph.0005748 (PMC12900349; doi:10.1371/journal.pgph.0005748)
Supplement: S2 Appendix — (DOCX) [file pgph.0005748.s002.docx]

## Structured Questionnaire

Emergency clinician awareness of healthcare entitlements and barriers to access for asylum seekers and refugees questionnaire

This questionnaire has been designed to evaluate nursing and medical staff awareness and knowledge of health entitlements and obstacles to obtaining good care for asylum seekers and refugees as part of a service evaluation hoping to improve awareness in the department. The questions are designed to gain an understanding of awareness across the department and is not to test you as an individual. It is not expected that you will be able to answer all the questions and any gaps in knowledge are useful for the development of teaching for everyone.

The data from this survey may be used internally within the department to develop teaching or resources to support you as a clinician. The data will be kept entirely anonymous and the only details we asking for is that which relate to your role. Participation is entirely voluntary and there is no obligation to complete the questionnaire. If you have any questions prior to completing this survey, please contact Daniel Dolan (Daniel.dolan@kcl.ac.uk/[Daniel.dolan@nbt.nhs.uk](mailto:Daniel.dolan@nbt.nhs.uk)) who is the lead researcher.

Please select your profession: medical/nursing/other (will redirect if chosen)

Please state your job title:

Please state your grade:

**Section A: Knowledge of Healthcare Entitlements and Barriers to Access for Asylum Seekers and Refugees**

Are you aware of any of the different immigration statuses under which migrants are classified by the UK Government?

1. Yes
2. No

How confident would you be to define the following statuses as per the UK Government immigration categories:

1. Not confident at all
2. Slightly confident
3. Somewhat confident
4. Fairly confident
5. Very confident
6. Asylum seeker
7. Refused asylum seeker
8. Refugee
9. Undocumented migrant
10. Limited leave to remain
11. Humanitarian protection

Are you aware of any recent changes to healthcare entitlements for asylum seekers and refugees? *

1 – Yes

2 – No

If yes, do you know where these changes effect within the UK? *

1 – UK-wide

2 – England only

3 – England and Wales only

4 – Scotland only

5 – England and Scotland only

Does immigration status affect what care someone can receive for free from the NHS?

1. Yes
2. No

Please add any comments:

How confident do you feel in your role as a clinician in determining what care a migrant is entitled to and whether it is free of charge?

1. Not confident at all
2. Slightly confident
3. Somewhat confident
4. Fairly confident
5. Very confident

Which of the following groups of patients are entitled to free emergency care in the UK (e.g in A&E)?*

a) Only people legally living in the UK or with leave to remain (i.e. not undocumented migrants or refused asylum seekers)

b) Asylum Seekers and Refugees (regardless of status), UK citizens, EU migrants

c) UK citizens only

d) Everyone, regardless of their immigration status

e) Don't know

Which groups of patients are entitled to free primary healthcare (e.g. registering at a GP)? *

a) Only people legally living in the UK or with leave to remain (i.e. not undocumented migrants or refused asylum seekers)

b) Asylum Seekers and Refugees (regardless of status), UK citizens, EU migrants

c) UK citizens only

d) Everyone, regardless of their immigration status

e) Don't know

Which groups of patients are entitled to free secondary healthcare (e.g. specialist clinic or admission to hospital)? *

a) Only people legally living in the UK or with leave to remain (i.e. not undocumented migrants or refused asylum seekers)

b) Asylum Seekers and Refugees (regardless of status), UK citizens, EU migrants

c) UK citizens only

d) Everyone, regardless of their immigration status

e) Don't know

What documents are required before registering someone at the GP? *

a) Passport or other form of photo ID

b) Birth certificate;

c) Proof of address;

d) None of the above;

e) All of the above.

f) Don't know

What documents are required before registering to be seen at an emergency department? *

a) Passport or other form of photo ID

b) Birth certificate;

c) Proof of address;

d) None of the above;

e) All of the above.

f) Don't know

**Section B: Barriers to access of Emergency Care**

How often do you ask patients about their immigration status?

1. Always
2. Often
3. Sometimes
4. Rarely
5. Never

Please add any comments:

Do you feel we should ask this routinely?

1. Definitely
2. Probably
3. Possibly
4. Probably Not
5. Definitely Not

Please add any comments:

If you were assessing a patient who was an asylum seeker or refugee, how likely would you be to ask them if they were registered at a GP?

1. Always
2. Often
3. Sometimes
4. Rarely
5. Never

Please add any comments:

If you do ask this question, how often do they state they are not registered with a GP?

1. Always
2. Often
3. Sometimes
4. Rarely
5. Never
6. I don’t ask

Please add any comments:

How often have you been aware of any migrants attending the department because they felt they were no entitled to care elsewhere (e.g. GP)

1. Always
2. Often
3. Sometimes
4. Rarely
5. Never

How often do you use a professional translation service or “language line”to communicate with patients who do not speak English as their first language?

1. Always
2. Often
3. Sometimes
4. Rarely
5. Never

How often do you use friends or family to communicate with patients who do not speak English as their first language?

1. Always
2. Often
3. Sometimes
4. Rarely
5. Never

How often do you use “google translate” or another online translation tool to communicate with patients who do not speak English as their first language?

1. Always
2. Often
3. Sometimes
4. Rarely
5. Never

How often do you feel cultural differences (for example, different expectations of how health systems work, or how to communicate in this environment or even cultural understanding of roles) affect the care you can provide to patients?

1. Always
2. Often
3. Sometimes
4. Rarely
5. Never

How often do you think immigration status affects our prescribing practices? (e.g. providing TTAs for medications that can be bought OTC)

1. Always
2. Often
3. Sometimes
4. Rarely
5. Never

From your experience, do asylum seekers and refugees have adequate arrangements for transport to and from there residence?

1. Definitely
2. Probably
3. Possibly
4. Probably Not
5. Definitely Not

**Section C: Local Context and Services**

Have you been aware of any changes to the numbers of ASRs attending the department?

1. Yes
2. No

If yes, are you aware of any factors influencing this change?

1. Yes
2. No

If yes please provide details:

From your experience, do you feel Bristol has adequate resources and services providing for asylum seekers and refugees?

1. Definitely
2. Probably
3. Possibly
4. Probably Not
5. Definitely Not

Are you aware of any specific services that you would recommend to your patients?

1. Yes
2. No

If yes, please provide details:

**Section D: Your opinion**

Would you be interested in engaging with education sessions/resources within the emergency department about asylum seeker and refugee health, entitlements and ways to improve their care?

1. Definitely
2. Probably
3. Possibly
4. Probably Not
5. Definitely Not

Please add comments:

What type of teaching would you be most interested to take part in, if any? (Select any that apply)

1. Formal training day
2. E-module
3. Brief sessions during departmental meeting
4. Microsoft teams teaching sessions
5. Education resources/guidance available on intranet/app,
6. other, please provide details:

Would it be useful to have education/resources within the department about local services available to asylum seeker or refugees and how to access them?

1. Definitely
2. Probably
3. Possibly
4. Probably Not
5. Definitely Not

Please add comments:

What type of resources would be most useful to help signpost patients to local services?

1. Information packs for patients
2. Online/app guidance for clinicians
3. Referral forms for local services (paper)
4. Referral forms for services (intranet/app
5. Other, please provide details:.

Have you got any suggestions on how education around this topic could be delivered?

Please state:

Are there any other ways you feel we could improve our signposting for asylum seeker or refugee patients to other local services?

Please state:

Are there any other ways you feel we could improve the quality of care we provide to asylum seeker or refugee patients?

Please State:

*Please note, this structured questionnaire was distributed using Microsoft Forms and final design can be accessed using the following link: https://forms.office.com/e/d025ucSXbp
