# Supplementary material for: A service evaluation of clinicians’ Signposting of asylum seekers and refugees (ASRs) attending an emergency department in South-West England
Source: PLOS Glob Public Health. 2026 Feb 12;6(2):e0005748. doi: 10.1371/journal.pgph.0005748 (PMC12900349; doi:10.1371/journal.pgph.0005748)
Supplement: S3 Appendix — (DOCX) [file pgph.0005748.s003.docx]

**INFORMATION SHEET FOR PARTICIPANTS**

**YOU WILL BE GIVEN A COPY OF THIS INFORMATION SHEET**

**Title of study**

**A Service Evaluation of clinicians’ knowledge of the entitlements, barriers to access to healthcare and appropriate signposting for Asylum Seekers and Refugees attending Southmead Emergency Department**

**Invitation Paragraph**

I would like to invite you to participate in this service evaluation which forms part of my masters research. Before you decide whether you want to take part, it is important for you to understand why the research is being done and what your participation will involve. Please take time to read the following information carefully and discuss it with others if you wish. Ask me if there is anything that is not clear or if you would like more information.

**What is the purpose of the study?**

The purpose of the study is to assess emergency clinicians’ awareness of entitlements and barriers to access of healthcare for asylum seekers and refugees and the local services that are available to them in order to inform specific interventions that can improve knowledge and signposting provided to this community.

**Why have I been invited to take part?**

You are being invited to participate in this study because you are a nurse or doctor working in Southmead Emergency Department. Although you may feel that assessing asylum seekers and refugees is not an integral part of your day-to-day work, I want to find out if there is any way I can help to support you to have the write education and tools to do this.

**What will happen if I take part?**

If you agree to take part you will complete a survey anonymously. The survey will ask you questions about you awareness of healthcare entitlements and barriers to access for asylum seekers and refugees, as well as what local services you are aware of and, lastly, if there is anything you feel could be done to support you to improve this knowledge. The survey will take you approximately 10 minutes to complete.

**Do I have to take part?**

Participation is completely voluntary. You should only take part if you want to and choosing not to take part will not disadvantage you in anyway. If you choose to take part you will be asked to provide your consent. To do this you will be asked to indicate that you have read and understand the information provided and that you consent to your anonymous data being used for the purposes explained.

You are free to withdraw at any point during completion of the survey, without having to give a reason. If you would like to withdraw please contact me directly ([Daniel.dolan@nbt.nhs.uk](mailto:Daniel.dolan@nbt.nhs.uk)). Withdrawing from the study will not affect you in any way. Once you submit the survey, it will no longer be possible to withdraw from the study because the data will be fully anonymous. Please do not include any personal identifiable information in your responses.

**What are the possible risks of taking part?**

There are no significant risks to you as a participant in this evaluation. It is important to state that this is not a test of you as an individual, instead it is an assessment of a service and what can be done to improve it. We do not expect you to know all the answers of information requested within this survey.

**What are the possible benefits of taking part?**

It is hoped that this evaluation will inform teaching, education and resources for you as a clinician working in the department to give you the support and information you need when assessing asylum seekers and refugees.

**Data handling and confidentiality**

This research is anonymous. This means that nobody, including the researchers, will be aware of your identity, and that nobody will be able to connect you to the answers you provide, even indirectly. Your answers will nevertheless be treated confidentially and the information you provide will not allow you to be identified in any research outputs/publications. Your data will be held securely on an encrypted drive until the final analyses are undertaken and report submitted.

**What will happen to the results of the study?**

The results of the study will be summarised in my report of this evaluation and my dissertation for my masters of public health. There will be no identifiable element and instead data will be shown in tables and graphs. If this evaluation is later seen to be of value then it may be submitted to a research/evaluation/quality improvement journal.

**Who should I contact for further information?**

If you have any questions or require more information about this study, please contact me using the following contact details:

Name: Daniel Dolan

Email: Daniel.dolan@kcl.ac.uk/daniel.dolan@nbt.nhs.uk

**What if I have further questions, or if something goes wrong?**

If this study has harmed you in any way or if you wish to make a complaint about the conduct of the study you can contact King's College London using the details below for further advice and information:

Name: Assaf Givati

Email: assaf.givati@kcl.ac.uk

**Thank you for reading this information sheet and for considering taking part in this research.**
