# Supplementary material for: A service evaluation of clinicians’ Signposting of asylum seekers and refugees (ASRs) attending an emergency department in South-West England
Source: PLOS Glob Public Health. 2026 Feb 12;6(2):e0005748. doi: 10.1371/journal.pgph.0005748 (PMC12900349; doi:10.1371/journal.pgph.0005748)
Supplement: Attachment — Submitted filename: Rebuttal letter PGPH-D-25-02560.docx [file pgph.0005748.s004.docx]

PLOS Global Public Health

PGPH-D-25-02560
A Service Evaluation of Clinicians’ Signposting* of Asylum Seekers and Refugees (ASRs) attending an Emergency Department in South-West England

A rebuttal letter that responds to each point raised by the editor and reviewers.

Thank you very much to the editor and the two reviewers for taking the time to review our manuscript, and for the thoughtful and helpful comments. The table below maps out the changes and amendments made in response to each comment:

| **Editor** | |
| --- | --- |
| **Comment** | **How this has been addressed** |
| 1. Please send a completed 'Competing Interests' statement, including any COIs declared by your co-authors. If you have no competing interests to declare, please state "The authors have declared that no competing interests exist". Otherwise please declare all competing interests beginning with the statement "I have read the journal's policy and the authors of this manuscript have the following competing interests:" | We added 'Competing Interests' Statement that "The authors have declared that no competing interests exist". |
| 2. Please amend your detailed Financial Disclosure statement. This is published with the article. It must therefore be completed in full sentences and contain the exact wording you wish to be published. If you did not receive any funding for this study, please simply state: “The authors received no specific funding for this work.” | We have added a financial disclosure statement: “The authors received no specific funding for this work”. |
| 3. We have noticed that you have uploaded Supporting Information files, but you have not included a list of legends. Please add a full list of legends for your Supporting Information files after the references list. | Full List on legends now placed after reference list.  It includes reference to the raw data which has been uploaded as supporting information. |
| 4. In the online submission form, you indicated that The data supporting a study's findings are not publicly accessible but can be obtained by other researchers if they make a reasonable request to the authors.  All PLOS journals now require all data underlying the findings described in their manuscript to be freely available to other researchers, either  1. In a public repository,  2. Within the manuscript itself, or  3. Uploaded as supplementary information.  This policy applies to all data except where public deposition would breach compliance with the protocol approved by your research ethics board. If your data cannot be made publicly available for ethical or legal reasons (e.g., public availability would compromise patient privacy), please explain your reasons by return email and your exemption request will be escalated to the editor for approval. Your exemption request will be handled independently and will not hold up the peer review process, but will need to be resolved should your manuscript be accepted for publication. One of the Editorial team will then be in touch if there are any issues. | We have uploaded the raw data for this evaluation as supplementary information. |
| 5. If the reviewer comments include a recommendation to cite specific previously published works, please review and evaluate these publications to determine whether they are relevant and should be cited. There is no requirement to cite these works unless the editor has indicated otherwise. | NA |
| 6. Please review your reference list to ensure that it is complete and correct. If you have cited papers that have been retracted, please include the rationale for doing so in the manuscript text, or remove these references and replace them with relevant current references. Any changes to the reference list should be mentioned in the rebuttal letter that accompanies your revised manuscript. If you need to cite a retracted article, indicate the article’s retracted status in the References list and also include a citation and full reference for the retraction notice. | The reference list had been reviewed and is up to date. |
| **Reviewer 1** | |
| **Comment** | **How this has been addressed** |
| I want to thank the authors for taking their interest on this important topic. In the last decade, the increasing number of ASR makes this topic of health service provision a very relevant one. The manuscript can be made stronger with a few adjustments. | Thank you very much. |
| 1. The manuscript is full of abbreviations, which makes the reading flow difficult. e.g UK, GP, SE, BMA, FY2-ST3, TTA, FP10, ED, NHS etc. Please have a look though out the manuscript and adjust accordingly. | We understand the issue and have significantly reduced the number of abbreviations, except for: UK, NHS and ASRs. |
| 2. The manuscript has a number of spelling mistakes, a copy editing process might be useful. Please check a few examples here with highlighted from the manuscript:   - Page 9 Evaluation phase (research design) please have a look at the second last sentence in this box, - Page 11 line 213, - page 10 in the table (The researcher) please check the second sentence in the last column. In the table (Outcome), please check the last sentence, - page 15, please check line 296, page 14, please check line 289. | The text has now been through copy editing (see comments in blue font)  Regarding each of the points:  Page 9 Evaluation phase (research design) please have a look at the second last sentence in this box – this has been removed.   - Page 11 line 213, - we tighten the sentence. - page 10 in the table (The researcher) please check the second sentence in the last column. – the sentence was removed. - In the table (Outcome), please check the last sentence, the sentence was removed - page 15, please check line 296, - the sentence was amended. - page 14, please check line 289. - the sentence was amended. |
| 3. Reference documents such as Safe Surgery tool kit and refugee and asylum seeker patient health tool kit could be provided as part of the annex or part of the supporting information. | The reference documents have now been included in the supporting information.  Please see list of legends, including *Doctors of the World* “Safe Surgeries” toolkit and *British Medical Association’s* Refugee and asylum seeker patient health toolkit. |
| 4. Under data analysis, the process and steps taken are clear for both quantitative and qualitative. However for the qualitative, it is important to highlight the five steps and the role of the second person for triangulation of information. | This has been added. |
| 5. Page 8, under ethical statement, do we have a similar response from Bristol Hospital as the Kings College London? | Please see added statement, lines 186-188: “The clinical effectiveness project application was submitted and approved by the Quality Governance Team of the NHS trust in which this project was undertaken on 16/12/2022 (Project application number CE50833).” |
| 6. Page 8 line 179, reads incomplete on its own. Considering this is also in the table, may useful to rewrite or remove it completely. | Sentence removed in place for reference to table 2. |
| 7. Page 9, please check if possible to increase the font size in the table. | Done. |
| 8. Page 10, you are repeating what is already stated on page 8, under ethics statement. | The repetition has been removed. |
| 9. Page 10, the statement in the table under knowledge and skills, It is important to focus on the skills of the research team, instead of writing or mentioning the supervisor, it is important to mention the skills of the supervisor (research team). | The table has been edited to read:  The lead researcher ensured adequate research of the topic including undertaking a systematic review prior to planning the present evaluation. Evaluation and research techniques employed were backed by evidence and with support of the dissertation supervisor who is experienced in research and publishing in the field of public and global health. |
| **Reviewer 2** | |
| **Comment** | **How this has been addressed** |
| An applaud to the authors for this interesting manuscript that focused on "A Service Evaluation of Clinicians’ Signposting* of Asylum Seekers and Refugees (ASRs) attending an Emergency Department in South-West England". | Many thanks. |
| Introduction:  Line 64: Consider changing “have” to “has” -Line 72 and throughout the manuscript: consider correcting “et al” to “et al.” -Line 105: For consistency, please check how Department is written in the lines above -Line 108: Consider changing “Clinician” to “Clinicians” -Line 151: Consider changing “recruited” to “recruitment” -Line 189: Table 2, under the section Measures taken for Research and design, consider changes “The contact details of the evaluation lead were be provided for any further questions about the evaluation” to “The contact details of the evaluation lead were provided for any further questions about the evaluation” | Line 64: Consider changing “have” to “has” - done  -Line 72 and throughout the manuscript: consider correcting “et al” to “et al.” - done -Line 105: For consistency, please check how Department is written in the lines above – done, now all are “Emergency Department”  -Line 108: Consider changing “Clinician” to “Clinicians” - done -Line 151: Consider changing “recruited” to “recruitment” - done -Line 189: Table 2, under the section Measures taken for Research and design, consider changes “The contact details of the evaluation lead were be provided for any further questions about the evaluation” to “The contact details of the evaluation lead were provided for any further questions about the evaluation” - done |
| Results: Given the small number of study participants relative to the countries population of medical staff, I would recommend that the authors should add the number of staff for each result i.e. x/x through the results. | Done. Proportions have been added for every result. |
| Results:  -Line 206: Consider moving the heading one line above - Line 213: delete duplicate word “that” -Line 241: Consider changing “using” to “use” -Line 258: Please check, either delete "and" or add what seems to be missing -Line 277: Consider moving this to two lines above -Line 289: Consider changing “acquire” to “enquire” -Line 325: remove the words “seen to be” -Line 354: Capitalize the word “Summary” Line 407-409: Inconsistency with word capitalization. Authors should stick to one style of writing reference titles. -Line 468: For consistency with other references, authors should consider capitalizing the letter after the colon (:). | -Line 206: Consider moving the heading one line above – done - Line 213: delete duplicate word “that” - done -Line 241: Consider changing “using” to “use” - done -Line 258: Please check, either delete "and" or add what seems to be missing - done -Line 277: Consider moving this to two lines above - done -Line 289: Consider changing “acquire” to “enquire” - done -Line 325: remove the words “seen to be” - done -Line 354: Capitalize the word “Summary” - done Line 407-409: Inconsistency with word capitalization. Authors should stick to one style of writing reference titles. - done -Line 468: For consistency with other references, authors should consider capitalizing the letter after the colon (:). - done |
